# Impact of Australian general practice alcohol and other drugs education: a qualitative evaluation

**DOI:** 10.3399/BJGPO.2022.0181

**Published:** 2023-04-19

**Authors:** Bryce Brickley, Lizette Fox-Miller, Paul Grinzi, Leigh Williams, Emily Lindsay, Shani Macaulay, Simon Slota-Kan

**Affiliations:** 1 Royal Australian College of General Practitioners (RACGP), East Melbourne, Victoria, Australia

**Keywords:** education medical, general practitioners, physicians general practice, physicians family, general practice, qualitative research

## Abstract

**Background:**

Many GPs are challenged to deliver safe and effective care for patients who use alcohol and other drugs (AOD). The Royal Australian College of General Practitioners (RACGP) developed the AOD GP Education Programme to support Australian GPs and optimise AOD care in the community. How the programme impacted GP participants is not yet fully understood.

**Aim:**

To explore the views and experiences of GP participants who completed the AOD GP Education Programme, and AOD experts who were involved in the programme as a presenter or mentor.

**Design & setting:**

Situated in the constructivist paradigm, this qualitive descriptive study engaged GPs across Australia.

**Method:**

This study employed semi-structured, online, focus groups interviews. Data were analysed thematically.

**Results:**

Five focus groups were held with a total of 35 GP participants. Five themes developed, which illustrated that the study participants viewed the programme design as comprehensive and flexible. It has also been shown that participants' individual learning needs were addressed. Impacts of the programme on clinical practice included the following: confidence to care for patients who use AOD; confidence to collaborate with colleagues in delivery of AOD care; confidence to develop AOD professional networks in their community setting; and confidence to manage complex AOD presentations.

**Conclusion:**

Participants described the AOD programme as a high quality and positive educational experience. The prioritisation of core treatment skills (whole-person care and structured approaches to behavioural change) was a feature of the professional development programme. The AOD programme design is a practical model to implement for future AOD GP education and continuing professional development.

## How this fits in

The traditional approach to GP AOD education is knowledge-based and does not optimally enable GPs to facilitate effective behavioural change. Practical skills-based GP strategies to support patients are lacking in current AOD education. This qualitative evaluation found that the RACGP AOD GP Education Programme was a high quality AOD educational experience for GP participants and trainers. Participants described beneficial impacts of the programme on their clinical practice, such as improved GP confidence to deliver care, and the development of professional relationships by collaborating in care. The RACGP AOD GP Education Programme design is a practical model for GP education and supports professional development by GPs.

## Introduction

AOD have a significant impact on the global burden of disease.^1^ The most investigated substance is alcohol, to which 1 in 20 of all deaths worldwide is attributable.^2^ Reducing AOD use overall improves social and emotional wellbeing, and reduces health risks within communities.^3^ In Australia, alcohol and illicit substances are collectively responsible for 7% of the national disease burden.^4^ The impacts of AOD use include exposure to violence, accidents, crime, injury, and housing difficulties. In addition, AOD use may lead to the development of chronic health conditions including cardiovascular disease, mental health problems, and dependency disorders.^4^ Effective intervention into AOD consumption is needed to support health and community wellbeing.

Approximately 90% of Australians visit a GP each year.^5^ Many Australians regularly consume AOD, with 82% of Australian adults reporting that they drink alcohol.^6^ The 2019 Australian National Drug Strategy Household Survey found the following: 25% 'binge' drink at least monthly; 16% have used an illicit drug in the past 12 months; and 4% have misused a prescription drug.^7^ Cannabis is the most commonly used illicit substance (37% reported use weekly or more often), followed by methamphetamine and amphetamine (17%), ecstasy (7%), and cocaine (5%).^7^ In Australia, GPs are embedded in the community and commonly see the health impacts of Australia’s high prevalence of AOD use.

Targeted health interventions delivered by GPs have been demonstrated to reduce AOD use and associated health risks in patients.^8^ However, GP-led AOD interventions are challenging to implement with success owing to factors such as lack of time and resources, consultation dynamics, and community stigmatisation of AOD use.^9–11^ Recent research has questioned the effectiveness of brief interventions for alcohol consumption.^9^ The provision of effective AOD care is influenced by GP attitudes, education, and role security.^10,11^ Patients typically present for reasons other than their substance use and will often not recognise the impact it has on their medical presentation (for example, a cardiovascular condition exacerbated by alcohol consumption).^10^ Initiating a conversation about substance use, then managing the outcomes of that conversation safely and effectively within the constraints of general practice, can be challenging for GPs.^12^

Australian GPs have access to an abundance of high quality, evidence-based educational resources about AOD. Most of these resources focus on clinical interventions for individual substances but are designed for specialist AOD services, rather than general practice.^13–15^ AOD GP education lacks practical strategies to support patients who present in primary care with complex needs, such as diverse comorbidities, and at various levels of preparedness to change. Furthermore, access to education may not address individual barriers to care such as lack of GP confidence and stigmatisation of patients.^16^

The RACGP, Australia’s largest general practice professional organisation, developed the AOD GP Education Programme (hereafter 'the programme') to increase GP confidence to address AOD use in patients. The programme design offered an innovative approach and aimed to deliver practical skills-based strategies, which were peer delivered ‘by GPs for GPs’. Strategies were based on whole-person care and the 5As (ask, assess, advise, assist, arrange) framework to structure patient consultations.^17–19^ The intention was to teach broadly applicable skills rather than specific substance and pattern of use approaches, with the aim of enabling programme participants to develop confidence in clinical strategies that are adaptable to their individual patients' needs. By using a strengths-based approach, the programme sought to equip GPs to confidently discuss AOD use with their patients, implement best-practice approaches, minimise harm, implement safer prescribing practices, and improve health.^20^

Approximately 3000 Australian GPs completed the programme between May 2020 and June 2022. Understanding participants’ perceptions and experiences will help inform future GP education, support clinical practice, and support advocacy for AOD healthcare priorities. This qualitative evaluation aimed to explore the views and experiences of those who engaged in any of the programme’s pathways (both funded and non-funded) and delivery formats (online learning modules, live online workshops, mentor-assisted training, and online case-based discussion).

## Method

### Research paradigm, qualitative approach, and research question

The purpose of this study was to evaluate the participant outcomes and impact of the programme. This study was situated in the constructivist paradigm,^21^ with a subjectivist epistemology, relativist ontology, and a naturalistic methodology.^22^ A qualitative descriptive approach^23^ facilitated an exploration of context and generated a rich description of experiences, feelings, and perspectives in the language of participants. The research question was as follows: 'What are the views and experiences of the RACGP AOD GP Education Programme by GP participants, presenters and/or mentors?'

### The programme overview

In 2019, the RACGP reached an agreement with the Australian Government Department of Health and Aged Care to deliver an education package that improves support and resourcing available for GPs to treat AOD-related problems. The RACGP designed and delivered an education programme with five key pathways, for which GPs were awarded continuing professional development points on completion. GPs could self-nominate to enter any programme pathway. These were:

1. **Essential skills training:** online self-directed education that introduced AOD care within Australian general practice (non-remunerated).
2. **Treatment skills training:** the programme was built around this pathway, which prioritised whole-person care across the 5As framework for behavioural change. Online live workshops (Zoom) and self-directed education focused on building core AOD consultation skills. Pathway completion attracted reimbursement of $1200 AUD.
3. **Advanced skills training:** mentor-assisted, personalised education for GPs seeking in-depth training to address AOD issues relevant to their local community. Pathway completion attracted reimbursement of $2500 AUD.
4. **AOD Connect: Project ECHO:** a national, virtual community of practice with GP-led case-based discussion, facilitated weekly by a panel of experts from various disciplines (non-remunerated).
5. **High-risk groups:** online self-directed education that emphasised whole-person care for patients at higher risk of harms associated with AOD use (for example, supporting people who have experienced trauma, people in contact with the criminal justice system, and Aboriginal and Torres Strait Islander peoples). This pathway was non-remunerated.

### Study design

This qualitative descriptive study employed semi-structured, online, focus groups interviews.^24^ This method helped enable time-limited clinicians from geographically dispersed areas to participate in the research.^24^ This study was conducted and reported in accordance with the 32-item consolidated criteria for reporting qualitative research.^25^

### Personal reflexivity statement

All researchers were RACGP employees at the time of the study. A collaborative approach helped mitigate any influence on the research by individual beliefs, expectations, and assumptions.^26^ The lead (BB) was the programme evaluation coordinator and experienced qualitative researcher. All other authors led the design and delivery of the programme. PG, SM, and SSK are practising GPs, educators, and AOD experts.

### Selection and recruitment of study population

Purposive sampling was conducted.^23^ Eligible participants were RACGP members in active clinical practice, who had completed one or more programme pathways, or were involved as a programme presenter or mentor. A sample size of 4–8 interviews^27^ was sought, with participants having diverse programme experiences and individual characteristics including age, career stage, and geography.

To identify GP participants, researchers (EL, LFM, LW) assessed completed post-training surveys of the online self-directed learning modules or live online workshops, and mentor post-training surveys. Responders who answered optional, open-text questions were identified for focus group recruitment. These participants demonstrated critical thinking and reflection of their programme experience. Frequent attendees of the weekly online case discussion groups were also identified. Email invitations were sent to identified participants. All identified participants were provided with an explanatory statement of the study in plain language, and an informed consent form. Participants were reimbursed $170 AUD on completion of the interviews.

### Focus group interview protocol

#### Preparation

Each focus group was intended to have 4–12 participants, and to involve participants with at least one homogenous characteristic (for example, GPs in remote and rural locations).^28^ Willingness to fully engage in a group discussion generates useful data and can be achieved more readily with a homogenous group.^29^ Interview facilitators were experienced GP medical educators, who were external to the programme. They were provided with the programme handbook before the interviews^30^ and were trained by the researchers on how to manage difficult participant behaviour, adopt neutral positionality in interviews, and understand their influence on participants.^26^

#### Protocol

Researchers (LFM, LW) identified eight important lines of inquiry and considered their importance within the characteristics of the sample (Supplementary file S1). The primary researcher (BB) developed a semi-structured interview guide using these concepts, which was reviewed and pilot tested within the research team (by BB, LW, EL). Testing enabled the tailoring of questions towards each group’s homogeneous characteristics (for example, Table 1). Participants were provided with the interview questions 1 week before the scheduled interview. Interview duration aimed to be 90 minutes. Using the interview guide, the facilitator posed questions, adding probing questions where possible to advance conceptual thinking and encourage participants to elaborate on initial ideas. A researcher (BB, EL, JAU) attended the interviews, made observations, and provided technical support to optimise group discussion.^31^

**Table 1.** Semi-structured interview guide (participants who completed online live workshops and online self-directed learning)

Inquiry purpose	Focus group question	Potential probing questions or topics	Estimated duration
Elicit GPs' **views and experiences** of the treatment skills programme.	Can you tell me about your experience of participating in treatment skills training?	Were there any advantages or disadvantages? Did it help you connect with your GP colleagues?	10 minutes
Explore participants’ **motivations for enrolment** into the treatment skills programme.	Why did you choose to enrol in the treatment skills programme?	What was the influence of the grant payment? If you did not enrol in other RACGP AOD programmes, why?	5 minutes
Explore the extent to which participants’ **learning needs** were met by the treatment skills programme.	Did the programme meet your learning needs? If so, how or if not, why not?	What were the main barriers and enablers, for example, access?	5 minutes
Explore perceived advancements of **clinical knowledge and skills** from the treatment skills programme.	How did the treatment skills programme develop your clinical knowledge and skills?	Can you describe influence on any specific skills or knowledge areas: for example, specific drugs, pharmacotherapy, counselling skills?	10–15 minutes
Explore impacts of the treatment skills programme on **clinical practice**.	Can you elaborate on any impacts of the treatment skills programme on clinical practice?	Are there any challenges to implementing learnings from the course in practice? What components of the programme were most helpful for you to support alcohol and other drug use in your practice? Describe a time where you drew on learnings or resources from the programme in your practice.	10–15 minutes
Describe participants’ perspectives of the **training model** delivered.	What are your views on the way the education was delivered?	What are your preferences for future training? Can you suggest any other alternative approaches?	10 minutes
Explore any suggested **modifications** to the treatment skills programme to inform future programmes.	If you were able to make any changes to the design of the AOD programme, what changes would you make?	Duration Engagement Resources Content	10 minutes
Provide an opportunity for participants to **raise any topics not yet discussed**.	Would you like to discuss any topics about the treatment skills programme, that we did not cover in today’s session?		5 minutes

AOD = alcohol and other drugs. RACGP = Royal Australian College of General Practitioners.

### Data collection

The following descriptive data were collected from the RACGP’s membership database: sex, age, career stage, country of medical qualification, and residential postcode. All other data collection and analyses were completed simultaneously. The interviews were conducted and recorded through videoconference (Zoom). Audio-recordings were transcribed verbatim via a paid transcription service, and researchers (BB, LW, EL) checked all transcripts for errors. All participants were invited to verify their transcripts before analysis.

### Data analysis

Participants’ postcodes were searched, and their rurality was classified by Modified Monash Model.^32^ Interview data were analysed using a constant comparative method and reflexive thematic analysis.^33^ De-identified transcripts were synthesised and imported into NVivo for coding and categorisation. Four researchers coded the data (LFM, BB, LW, EL). To support inter-rater reliability, the lead researcher (BB) trained all coders before the analytical phase. Researchers developed codes in an open, data-driven approach.^34^ Codes were organised by the eight important lines of inquiry (Supplementary File S1). The analytical process was highly reflective, aided by field notes, and the entire research team took part in reviewing, defining, and naming themes.^35^ Researchers met weekly to debrief throughout the entire analytical phase. After the analysis of data from five interviews, consensus on data saturation was reached and further data collection was not required.^36^

## Results

### Focus group interviews and participants

Sampling occurred between October 2021 and March 2022. A total of 386 program participants, 65 mentors, and 23 presenters and/or subject matter experts were identified for recruitment. Of these, 379 were invited to participate, and thirty-five GP participants including presenters and mentors were interviewed. Their individual characteristics are shown in Table 2.

**Table 2.** Participants’ individual characteristics

Focus group interview	Duration, mins	Participants (*n* = 35)	Sex	Age, years	Career stage^a^	Country of university medical qualification	MMM classification^b^
AOD expert trainers and mentors	84	6 (GP1–6)	F = 4 M = 2	Mean: 58 Range: 45–67	Late career = 1 Pre-fellowship = 2 Mid-career = 3	Australia = 4 India = 1 UK = 1	MM 1 = 4 MM 2 = 1 MM 3 = 1
GPs who completed training via online live workshops and online self-directed learning	81	8 (GP7–14)	F = 6 M = 2	Mean: 43 Range: 31–68	Mid-career = 5 New fellow = 3	Australia = 6 NR (not Australia) = 1 Taiwan = 1	MM 1 = 4 MM 2 = 3 MM 3 = 1
GPs who completed case-based discussion and multiple training modalities	84	9 (GP15–23)	M = 7 F = 2	Mean: 52 Range: 39–65	Mid-career = 7 Pre-fellowship = 2	Australia = 4 Japan = 1 NR (not Australia) = 2 UK = 1 Venezuela = 1	MM 1 = 8 MM 3 = 1
International medical graduates and GP registrars	78	7 (GP24–30)	F = 4 M = 3	Mean: 42 Range: 29–53	New fellow = 4 Pre-fellowship = 3	Australia = 3 India = 1 Iraq = 1 Nigeria = 1 Sri Lanka = 1	MM 1 = 2 MM 3 = 3 MM 4 = 2
GPs in rural and remote locations	80	5 (GP31–35)	F = 5	Mean: 50 Range: 32–69	Mid-career = 4 New Fellow = 1	Australia = 1 India = 1 NR (not Australia) = 1 UK = 1 US = 1	MM 4 = 2 MM 5 = 3

^a^Career stage: pre-fellowship, any member who has not yet reached fellowship; new fellow, any member with fellowship within the past 5 years; mid-career, any fellow for >5 years but <35 years; late career; any fellow for >35 years. ^b^Modified Monash Model classifications: 1, metropolitan areas; 2, regional centres; 3, large rural towns; 4, medium rural towns; 5, small rural towns.

AOD = alcohol and other drugs. F = female. M = male. MM = Modified Monash category. MMM = Modified Monash Model. NR = not reported.

A different interview guide was developed for each of the following groups sharing a homogenous characteristic: (1) AOD expert trainers and mentors; (2) GPs who completed training via live online workshops and self-directed online learning; (3) GPs who completed case-based discussion and multiple training modalities; (4) international medical graduates and GP registrars; and (5) GPs in rural and remote locations.

### Reflexive thematic analysis

The following five themes developed: (1) empowering GPs to address a variety of gaps in knowledge, skills, and confidence to treat AOD; (2) understanding and addressing the needs of GP participants; (3) delivering an accessible, high quality professional development experience; (4) optimising whole-person approaches to AOD care to support behaviour change; and (5) learning alongside colleagues, and developing professional relationships to enhance AOD care. The themes are described below with illustrative quotations.

#### Theme 1: Empowering GPs to address a variety of gaps in knowledge, skills, and confidence to treat AOD

The programme empowered GPs to develop skills, knowledge, and confidence to deliver AOD care that addressed their identified learning opportunities. One GP enrolled in the programme to increase their overall AOD knowledge and skills, and stated *'with alcohol and other substances* [...] *I had no idea where to start or what to do, so that’s why I signed up*' (GP8). Another GP identified the need to improve their confidence to treat alcohol use, which was exacerbated by widespread alcohol consumption in their community; *'... there’s so much alcohol intake within the rural community of Australia that is completely … out of my comfort zone*' (GP32). Another GP self-identified the need to develop skills to establish the therapeutic alliance and effectively manage AOD conversations:

*'... there was a doctor who had left* [a nearby practice] *…. a lot of the patients started coming to see me, and some of the doses of this medications I was seeing, they were really scary … it was more how to present it* [safer doses and treatments] *to the patient, not necessarily the knowledge-base.'* (GP28)

One GP’s reason for enrolling demonstrated their continuing commitment to their patients:

*'... a couple of my long-term patients had disclosed to me their alcohol use … I felt completely unable and had no skills to help them … so I thought it was a great time to pick up some skills for these two patients.'* (GP20)

#### Theme 2: Understanding and addressing the needs of GP participants

Participants discussed needs as learning needs (for example, desire for knowledge about specific substances); learning preferences (for example, case-based learning); and care needs of their community setting (for example, prevalence of substance use). A wide range of learning needs among programme participants was recognised by one GP trainer:

*'There is an incredible diversity of learning needs. Some people came into* [the programme] *that* [were] *doing a lot of AOD medicine. We've had other people who did extremely little and had every shade of grey in between.'* (GP3)

The education design was important in addressing the wide range of needs and preferences of participants. Most participants expressed that the programme addressed their individual needs; for example, one GP said:

*'I feel that it was fulfilling all my needs which is required by me taking care of the patients who are coming to me … It seemed to me as the right pieces of information …'* (GP25)

The inclusion of structured approaches to specific treatments in general practice, such as safe home-based alcohol withdrawal, were vital to meeting GP learning needs. One GP attributed this approach to an increase in confidence:

*'There was a really good module about alcohol withdrawal and step-by-step … that was really well done and that gave me more confidence, especially having that scoring system to know when it’s safe to withdraw …*' (GP27)

Participants valued the case-based learning format, with case examples across a variety of substance presentations, degrees of complexity, and readiness to change. One participant emphasised the value of GP-led case-based learning:

*'The great strength of this* [programme] *is the inclusion of the cases … there’s nothing to be lost by adding more cases and perhaps they are optional extras. Maybe you don’t need to engage with every single one if that’s something that you* [don’t] *need at the time.'* (GP29)

Participants recognised that their needs evolved over time. One GP reflected that the programme encouraged them to seek further professional development:

*'Yes, it* [the programme] *did meet my learning needs at the time … it encouraged me to go on and learn more … I did end up spending a bit of time in a drug and alcohol unit.'* (GP29)

#### Theme 3: Delivering an accessible, high quality professional development experience

Overall, participants’ views of the programme were positive. However, study participants' experiences were highly individual because they described engaging with the programme in different ways, such as in weekly case-based discussion, mentor-assisted training, or online self-directed education. Many considered that the programme was a highly accessible, high quality educational experience, and they described important components that contributed towards this. Choice and flexibility meant that GPs were empowered to control their experience. This was particularly evident for the self-directed training, which some GPs completed over a period of months:

*'… I really liked the self-directed aspect ‘cause I’m a mum, I was in very early stages of pregnancy, I was pretty sick. It was great, I could just sit on my couch and do some learning, it’s perfect when I felt well enough to … I liked that it gave me some extra time to be able to look up stuff that I found really interesting or was a deficit in my knowledge … I did find it very isolating though and that would be the main disadvantage. I had no one to bounce ideas off, ask questions, or hear those really interesting stories.'* (GP7)

While self-directed training offered limited opportunities to connect with colleagues, some GPs completed the course alongside colleagues, which supported their learning, for example:

*'… Some colleagues around me were also doing it, so we sort of did it together ….' (*GP29)

GPs valued the ability to implement new learnings and practical strategies in real time, when treating AOD in practice. One GP said:

*'… during this program, I really utilised my patients, my patient problems, the real problems and I try to seek answers through what I learnt.'* (GP24)

Reimbursement helped participants meaningfully engage with the content. It encouraged GPs to place higher value on the opportunity and made them feel more valued, particularly in the context of fee-for-service remuneration with high levels of non-billable working hours. Conversely, the absence of funding for AOD professional development was thought to hinder GP engagement. Two participants stated:

*'We do so much unfunded time and CPD* [continuing professional development] *there is no way I would have committed to so many hours if it wasn’t funded.'* (GP34)

*'... being paid for a course really makes me value it a lot more. Made me feel like I’m committed to this … time like I want to get something out of it … I do so many things that are unpaid, that being paid for something really makes you feel a bit more valued and want to be a bit more interested in the area.'* (GP14)

#### Theme 4: Optimising whole-person approaches to AOD care to support behaviour change

**Figure 1.**
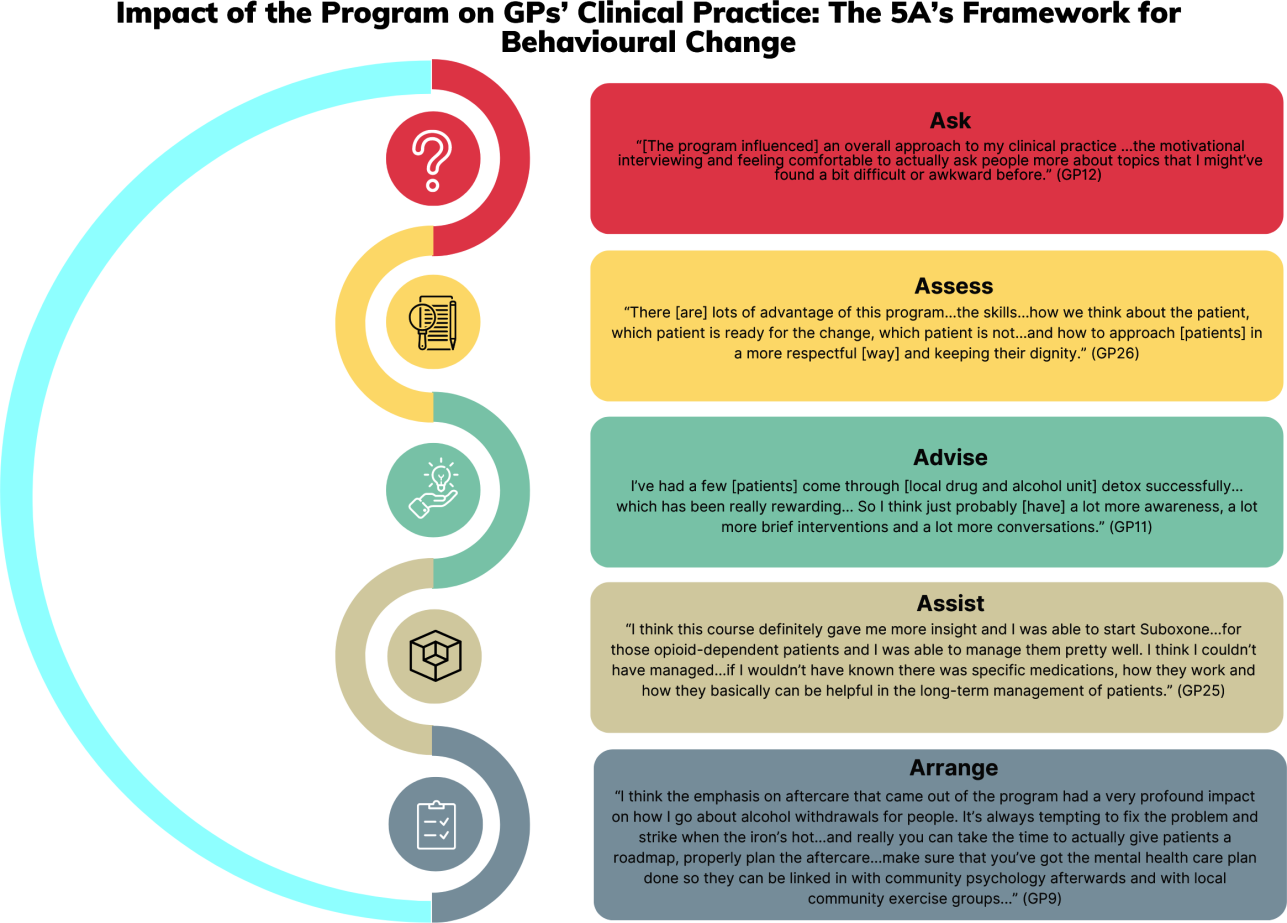
Impacts of the AOD programme on GPs’ clinical practice: the 5As framework for behavioural change.

The programme enabled GPs to develop a broad range of AOD clinical skills to facilitate patient behavioural change. Many GPs described the most significant development of clinical skills in relation to the 5As framework, such as to *‘assist’* patients with their AOD use. Overall, GPs described that the programme improved AOD confidence, skills, and knowledge, which influenced patient care across the 5As for a range of commonly used substances (Figure 1). One GP described the influence of the programme on self-confidence to plan long-term care for patients with chronic substance use:

*'*[the programme] *certainly gave me confidence in managing chronic cannabis use in the community … and put together a plan for weaning and cessation and mapping that out for patients.'* (GP9)

The programme assisted GPs to optimise their AOD care by utilising a whole-person care approach. GPs described greater confidence to explore their patients’ psychological and social wellbeing, which helped foster better connection and support patient autonomy:

*'I’ve learnt too that there is a need … to establish connection between current illnesses and the psychosocial, if there are any background issues which can influence* [their AOD] *use.'* (GP18)

*'It makes us more confident to know how to do a patient-centred conversation about addiction and help them with their need, not what we want to do for them.*' (GP26)

#### Theme 5: Learning alongside colleagues and developing professional relationships to enhance AOD care

Many participants described how they learnt alongside colleagues and developed professional relationships during and after the programme. This enabled GPs to learn from the experiences, perspectives, and questions of other programme participants and GP and/or specialist presenters and mentors. While engagement with colleagues was described across all focus groups, it was emphasised by those who had engaged with the online case-based discussion group (named Project ECHO). One participant viewed this community of practice as a clinical safety net: *'... having ECHO as a safety person or group of people I could talk to' (GP20*). Connecting with colleagues helped reduce feelings of isolation among participants, as shown by one GP who reflected on the impact of hearing other GPs’ AOD care experiences:

*'I didn’t know much, and I was sometimes feeling like I’m out on my own. It was great to be able to hear what others do and go, "That’s right. I’ve encountered that. What did I do before? How can I be doing it better?" So I found that really useful.'* (GP14)

The programme gave some GPs greater confidence, tools, and knowledge to act on AOD issues in their local practice, and actively share the programme’s resources and learnings with their colleagues. One GP supported a GP colleague with safer prescribing:

*'I see some of his patients and I see their opioid use … I’ve been able to educate him on how he can speak with them in a non-confrontational way … my colleague also benefited from* [the programme]*.'* (GP28)

The programme helped GPs to develop a greater understanding of support services and referral pathways that were available to them. One GP said: *'It* [the programme] *has led to greater collaboration of care with our local AOD workers within our community, which has been good' (GP9*). Participants felt empowered to engage and explore AOD clinical advisory services:

*'I was doing the course at home,* [at] *around 10 or 11pm, I saw this number that says it’s the hotline to call for someone who is withdrawing … I remember calling the alcohol hotline and I had a very good conversation with a lady who picked up the phone for about 30 minutes. It added to my education, and it made me confident to know that it’s not just "one of those numbers". It’s a number that works.'* (GP28)

Since completing the programme, participants reported receiving more referrals of patients needing support for AOD use. One GP felt that by completing the programme, their AOD clinical capabilities were affirmed to their practice team and community. He said: *'After doing the course … my colleagues are more confident that I can take care of these kind of patients...*' (GP25).

## Discussion

### Summary

This study explored the experiences and perceptions of GPs, presenters, and mentors participating in the RACGP AOD GP Education Programme. Overall, study participants viewed the programme to be a high quality educational experience that addressed their individual learning needs. Improved confidence, skills, and capacity to deliver AOD care for a range of substances and complex patient presentations were highlighted by GP participants. Since completing the programme, participants described confidently initiating conversations about AOD use, listening to patients, following a structured approach to care, sharing knowledge with their GP colleagues, coordinating care, and working with AOD specialists and other support services. The analyses will inform future GP education and professional development, and support GP-led AOD care in clinical practice.

### Strengths and limitations

This study was conducted by a multidisciplinary team that included programme staff, practising GPs, and medical educators. Researchers were trained and participated in frequent debriefing, which supported trustworthiness, credibility, and dependability of the analyses.^37^ This collaboration utilised the strengths of each researcher while minimising any influence of the researchers’ clinical presuppositions and programme-related experiences on the work.^21^ The rigorous approach, and commitment to transparency and replicability are evidenced through adherence with the COnsolidated criteria for REporting Qualitative research (COREQ) checklist.^25^

The sampling strategy supported recruitment within programme evaluation deadlines. The sample was highly diverse, with varied clinical experience, age, practice contexts, and involvement in the programme. However, the sampling approach is a notable limitation because it omitted the patient perspective and may have led to the recruitment of GPs with a more developed interested in AOD, medical education, and/or strong views about the programme. Potential GP participants were selected based on if they completed surveys, and provided a positive, negative, or neutral view, and were not recruited on the nature of the view they expressed. This approach sought to support the dependability of the analyses by establishing a sample of GPs who had already reflected on their program experience The patient perspective was out of scope of the present evaluation because the evaluation resourcing and timeline did not enable the engagement of patients as partners in the programme evaluation.^38^ Future GP education programme evaluations should include adequate time and resourcing to engage patients as equal and ongoing partners throughout the programme design, delivery, and evaluation.^38^

The analysis describes the views and experiences of study participants. Focus group interviews can generate descriptive data of participants’ experiences, thoughts, and feelings within a specific context.^39^ The transferability of the analyses to other health systems may be limited because the Australian general practice context will differ from other countries.^29,40^

### Comparison with existing literature

NHS Health Scotland (now Public Health Scotland) delivered a national training programme that aimed to enhance the capacity of GPs to deliver alcohol brief intervention (ABI).^41^ The programme found it challenging to meet the diverse needs of GPs and general practice teams across Scotland.^41^ The present study's analyses support that GPs’ learning needs are highly individual and diverse; however, the flexibility of the programme design enabled most GPs to maintain autonomy and agency during their educational experience. Strategies to cater for a wide variety of GP needs and preferences were strengths of the programme and should be considered in the design and delivery of future GP education programmes.

Study participants described structured approaches (for example, the 5As framework) and the prioritisation of whole-person care to support confidence to manage AOD across a variety of substances. In line with this, the Scottish ABI programme identified that to positively influence GP care, a holistic approach to patient care was essential.^41^ Several GPs in the present study described impacts including greater knowledge, clinical processes, and new relationships within their local practice and community. This suggests that the programme has helped GPs address barriers to AOD treatment that occur within patient care (for example, GP knowledge and confidence), the practice context (for example, clinical processes), and the wider community setting (for example, coordinating care).^42^ Screening, Brief Intervention, and Referral to Treatment (SBIRT) when implemented by GPs, has been found to address common AOD treatment barriers.^43^ SBIRT utilises a structured approach to care underpinned by substance use awareness and behavioural change counselling.^43^ The clinical framework of a structured approach incorporating whole-person care assists GPs to facilitate and manage care, and clarify links between general practice and specialist AOD services.

### Implications for research and practice

The successes of this programme can inform a new paradigm of strengths-based GP education to support clinical practice. A strengths-based approach to GP training should be GP-centric, recognising the diversity of GPs’ needs. Multiple educational modalities and remuneration are important design elements for addressing common barriers for GP engagement in professional development, such as a lack of time and access.

Improvements to clinical practice were described by programme participants as a direct result of engaging with the educational programme, which included greater confidence to explore psychological and social wellbeing, deliver care across the 5As framework, support patient autonomy, and plan long-term care. These benefits relate to one’s awareness, understanding, and confidence to deliver whole-person care and core consultation skills. Improved foundational treatment knowledge and skills are likely to lead to sustained benefits owing to the high applicability and regularity of their use across multiple domains in primary care. However, the sustainability of any programme benefits cannot be known from this cross-sectional study and should be further investigated. Furthermore, some programme participants in the study described benefits in skills and confidence to manage complex AOD treatment, such as with the management of a GP-led home-based alcohol withdrawal, and pharmacotherapy for opioid dependence. However, influence of the programme on the care of patients from priority populations who are disproportionately impacted by AOD use (for example, Aboriginal and Torres Strait Islander peoples)^7^ was not explicitly described by participants. Further evaluation is needed to explore the extent to which the programme and its approach influenced AOD use among priority patient populations.

In conclusion, the perceptions and experiences of participants of the RACGP AOD GP Education Programme indicates an increased confidence and capacity to treat AOD resulting from programme participation. The success of the programme stemmed from its design (‘by GPs for GPs’), theoretical scaffolding for behaviour change, structured approaches, whole-person care, flexibility, comprehensiveness, and remuneration. Future work needs to ensure that design and delivery of professional development is GP-led to successfully help other GPs navigate the complexity of AOD education and treatment.

## Data

The dataset relied on in this article is available from the corresponding author on reasonable request.
